# Intake Patterns of Specific Alcoholic Beverages by Prostate Cancer Status

**DOI:** 10.3390/cancers14081981

**Published:** 2022-04-14

**Authors:** Hui-Yi Lin, Tung-Sung Tseng, Xinnan Wang, Zhide Fang, Arnold H. Zea, Liang Wang, Julio Pow-Sang, Catherine M. Tangen, Phyllis J. Goodman, Alicja Wolk, Niclas Håkansson, Manolis Kogevinas, Javier Llorca, Hermann Brenner, Ben Schöttker, Jose Esteban Castelao, Manuela Gago-Dominguez, Marija Gamulin, Davor Lessel, Frank Claessens, Steven Joniau, the PRACTICAL Consortium, Jong Y. Park

**Affiliations:** 1 Biostatistics Program, School of Public Health, Louisiana State University Health Sciences Center, New Orleans, LA 70112, USA; xinnan.wang@alumni.lsuhsc.edu (X.W.); zfang@lsuhsc.edu (Z.F.); 2 Behavioral and Community Health Sciences Program, School of Public Health, Louisiana State University Health Sciences Center, New Orleans, LA 70112, USA; ttseng@lsuhsc.edu; 3 Department of Microbiology, School of Medicine, Louisiana State University Health Sciences Center, New Orleans, LA 70112, USA; azea@lsuhsc.edu; 4 Stanley S. Scott Cancer Center, Louisiana State University Health Sciences Center, New Orleans, LA 70112, USA; 5 Department of Tumor Biology, Moffitt Cancer Center & Research Institute, Tampa, FL 33612, USA; liang.wang@moffitt.org; 6 Department of Genitourinary Oncology, Moffitt Cancer Center & Research Institute, Tampa, FL 33612, USA; julio.powsang@moffitt.org; 7 SWOG Statistical Center, Fred Hutchinson Cancer Research Center, Seattle, WA 98109, USA; ctangen@fredhutch.org (C.M.T.); pgoodman@fredhutch.org (P.J.G.); 8 Department of Surgical Sciences, Uppsala University, SE-751 85 Uppsala, Sweden; alicja.wolk@ki.se; 9 Unit of Cardiovascular and Nutritional Epidemiology, Institute of Environmental Medicine, Karolinska Institutet, SE-171 77 Stockholm, Sweden; niclas.hakansson@ki.se; 10 Barcelona Institute of Global Health (ISGlobal), 08036 Barcelona, Spain; manolis.kogevinas@isglobal.org; 11 IMIM (Hospital del Mar Medical Research Institute), 08003 Barcelona, Spain; 12 Universitat Pompeu Fabra (UPF), 08002 Barcelona, Spain; 13 CIBER Epidemiología y Salud Pública (CIBERESP), 28029 Madrid, Spain; javier.llorca@unican.es; 14 University of Cantabria, 39005 Santander, Spain; 15 Division of Clinical Epidemiology and Aging Research, German Cancer Research Center (DKFZ), D-69120 Heidelberg, Germany; h.brenner@dkfz.de (H.B.); b.schoettker@dkfz.de (B.S.); 16 German Cancer Consortium (DKTK), German Cancer Research Center (DKFZ), D-69120 Heidelberg, Germany; 17 Division of Preventive Oncology, German Cancer Research Center (DKFZ) and National Center for Tumor Diseases (NCT), Im Neuenheimer Feld 460, D-69120 Heidelberg, Germany; 18 Genetic Oncology Unit, CHUVI Hospital, Complexo Hospitalario Universitario de Vigo, Instituto de Investigación Biomédica Galicia Sur (IISGS), 36204 Vigo, Spain; esteban.castelao@med.usc.edu; 19 Genomic Medicine Group, Galician Foundation of Genomic Medicine, Instituto de Investigacion Sanitaria de Santiago de Compostela (IDIS), Complejo Hospitalario Universitariode Santiago, Servicio Galego de Saúde, SERGAS, 15706 Santiago de Compostela, Spain; mgago@med.usc.edu; 20 Department of Family Medicine and Public Health, Moores Cancer Center, University of California San Diego, La Jolla, CA 92037, USA; 21 Department of Oncology, University Hospital Centre Zagreb, 10 000 Zagreb, Croatia; mgamulin8@gmail.com; 22 School of Medicine, University of Zagreb, 10 000 Zagreb, Croatia; 23 Institute of Human Genetics, University Medical Center Hamburg-Eppendorf, D-20246 Hamburg, Germany; d.lessel@uke.de; 24 Molecular Endocrinology Laboratory, Department of Cellular and Molecular Medicine, Campus Gasthuisberg, University of Leuven, Herestraat 49, P.O. Box 901, 3000 Leuven, Belgium; frank.claessens@med.kuleuven.be; 25 Department of Urology, University Hospitals Leuven, Herestraat 49, P.O. Box 7003 41, 3000 Leuven, Belgium; steven.joniau@uzleuven.be; 26 The Prostate Cancer Association Group to Investigate Cancer Associated Alterations in the Genome Consortium, Sutton SM2 5NG, UK; practical@icr.ac.uk; 27 Department of Cancer Epidemiology, Moffitt Cancer Center & Research Institute, Tampa, FL 33612, USA; jong.park@moffitt.org

**Keywords:** alcohol, beverage, prostate cancer, aggressiveness

## Abstract

**Simple Summary:**

Previous studies have shown heavy intake of different alcoholic beverages affects prostate cancer (PCa) clinical outcomes differently. However, the intake patterns of specific alcoholic beverages for PCa status are understudied. The study’s objective is to evaluate intake patterns of total alcohol and three types of alcoholic beverage (beer, wine, and spirits) by PCa risk and aggressiveness status. This study included 10,029 men with European ancestry (4676 non-PCa men and 5353 PCa patients). We found PCa patients had a similar total heavy alcohol intake compared with non-PCa men. However, PCa patients were likely to drink more wine and spirits than non-PCa men. Patients with aggressive PCa drank more beer but not wine and spirits. Interestingly, heavy wine intake was inversely associated with PCa aggressiveness. These findings suggest that the intake patterns of specific alcoholic beverages differ by PCa status, and this information might help develop personalized alcohol intervention for PCa patients.

**Abstract:**

Background: Previous studies have shown that different alcoholic beverage types impact prostate cancer (PCa) clinical outcomes differently. However, intake patterns of specific alcoholic beverages for PCa status are understudied. The study’s objective is to evaluate intake patterns of total alcohol and the three types of beverage (beer, wine, and spirits) by the PCa risk and aggressiveness status. Method: This is a cross-sectional study using 10,029 men (4676 non-PCa men and 5353 PCa patients) with European ancestry from the PCa consortium. Associations between PCa status and alcohol intake patterns (infrequent, light/moderate, and heavy) were tested using multinomial logistic regressions. Results: Intake frequency patterns of total alcohol were similar for non-PCa men and PCa patients after adjusting for demographic and other factors. However, PCa patients were more likely to drink wine (light/moderate, OR = 1.11, *p* = 0.018) and spirits (light/moderate, OR = 1.14, *p* = 0.003; and heavy, OR = 1.34, *p* = 0.04) than non-PCa men. Patients with aggressive PCa drank more beer than patients with non-aggressive PCa (heavy, OR = 1.48, *p* = 0.013). Interestingly, heavy wine intake was inversely associated with PCa aggressiveness (OR = 0.56, *p* = 0.009). Conclusions: The intake patterns of some alcoholic beverage types differed by PCa status. Our findings can provide valuable information for developing custom alcohol interventions for PCa patients.

## 1. Introduction

Prostate cancer (PCa) is the second most common cancer for men worldwide, accounting for 7.3% of cancer incidence and 3.8% of cancer deaths [1]. In 2020, globally, approximately 1.4 million men were diagnosed with PCa, and ~375,000 died due to PCa [1]. A growing number of studies reported that excessive alcohol intake is associated with a high PCa risk and aggressiveness [2,3,4,5]. A meta-analysis study including 27 studies observed a significant positive dose-response relationship between the level of alcohol intake and PCa risk starting with a low volume of alcohol intake [2]. Another meta-analysis with 43 studies showed that alcohol use is positively associated with the risk of overall PCa [6]. Moreover, a large cohort study with ~294,000 US men aged 50–71 showed that the amount of alcohol consumed daily was associated with non-advanced PCa risk [7]. However, other studies reported no significant associations between alcohol intake and PCa risk [8,9,10].

Although the causal relationship between excessive alcohol intake and PCa risk/aggressiveness is inconclusive [2], it is commonly accepted that excessive alcohol intake is not recommended for cancer survivors. Based on the 2020–2025 United States (US) Dietary Guidelines for Americans, this guideline suggested people who have certain medical conditions or are taking certain medications that can interact with alcohol should not drink alcohol at all [11]. Therefore, cancer survivors should avoid alcohol intake because they qualify for both conditions. Contrary to belief, PCa survivors have a similar excessive alcohol intake pattern compared to the general non-cancer male population based on a US population-based study [12]. The prevalence of frequent alcohol intake (light/moderate/heavy) during the past year is similar between non-cancer individuals (52.2%) and PCa survivors (51.3%). Additionally, the prevalence of heavy alcohol intake is almost the same (5.2% vs. 4.2%) shown in the 2012–2017 National Health Interview Survey, a US population-based survey [12]. The same study showed that heavy alcohol intake status prevalence is similar regardless of cancer status and length of cancer history after adjusting for other factors [12].

Associations between excessive alcohol intake and PCa clinical outcomes are inconsistent across studies. These conflicting findings may be due to many reasons, including over-generalization of all types of alcoholic beverages and various alcohol measurements of the complicated alcohol patterns [2,3,13]. As an example, most current PCa studies focused on total alcohol intake regardless of specific alcoholic beverage types. However, evaluations of total alcohol intake may not be a good approach in PCa studies because it has been shown that different types of alcoholic beverages may have a different impact on PCa risk and aggressiveness [3,4]. For example, high consumption of total alcohol was shown to be associated with a higher risk of high-grade PCa (odds ratio [OR] = 1.40), and this association was majorly driven by beer intake based on a population-based study [3]. Nevertheless, alcohol intake patterns of various beverage types by PCa risk and aggressiveness status are understudied. To address this issue, this study aims to evaluate alcohol intake patterns in terms of drinking frequency of total alcohol and the three beverage types (beer, wine, and spirits) by PCa risk and aggressiveness status.

## 2. Materials and Methods

### 2.1. Study Population

In this cross-sectional study, we included 4676 non-PCa men and 5353 PCa patients (including 837 (15.7%) patients with aggressive PCa) in the OncoArray project in the Prostate Cancer Association Group to investigate Cancer Associated Alterations in the Genome (PRACTICAL) Consortium, a large international prostate cancer collaborative group. The eligibility criteria of this alcohol study are men with European ancestry, valid PCa information, and current alcohol intake status (Figure S1). The majority of participants in the PRACTICAL consortium had European ancestry, so other races were excluded from this study. This study took advantage of the rich source of alcohol intake data in the OncoArray project, which was designed to evaluate genetic variants for association with the risk of PCa using a custom single nucleotide polymorphism (SNP) genotyping array. The details of this OncoArray project can be seen on the PRACTICAL Consortium website (http://practical.icr.ac.uk, accessed on 28 February 2022). The ancestry analyses were performed based on 2318 ancestry-related SNPs using principal component analysis. Men with European ancestry were defined as men with an estimated proportion of European ancestry > 80% based on the first two principal components. The details of ancestry analyses can be reviewed in the previous publication [14].

### 2.2. Measurements

PCa aggressiveness is defined as patients with a Gleason score ≥ 8, PSA > 100 ng/mL, ‘distant’ disease stage, or PCa-specific death. The alcohol intake information was collected using questionnaires or interviews (see Table S1). For having consistent reference period of alcohol intake, only participants with valid information of current alcohol intake were included. For alcohol, frequencies of current intake of three specific alcoholic beverage types (beer, wine, and spirits) were collected based on the following 10 categories: never or less than once a month, 1–3 times per month, once a week, 2–4 times per week, 5–6 times per week, once a day, 2–3 times per day, 4–5 times per day, 6+ times per day, and unknown. In this study, we categorized alcohol frequency intake patterns into three groups (infrequent, light/moderate, and heavy intake) based on the concept of the US National Health Interview Survey (NHIS) [15]. ‘Infrequent’ drinking was defined as never or less than once a month, ‘light/moderate’ drinking was defined as ≥1 time monthly to <2 times per day, and ‘heavy’ drinking was defined as ≥2 times per day. ‘Total’ alcohol intake status took all three beverage types (beer, wine, and spirits) into consideration, so only men with valid responses for all three specific beverage questions were included for the total alcohol measure. Heavy intake of total alcohol was defined as having heavy intake for any beverage types, and infrequent drinking for total alcohol was defined as an irregular intake for all three alcoholic beverage types. The body mass index (BMI) was categorized into three categories: normal/underweight (BMI < 25), overweight (BMI: 25–29.9), and obesity (BMI ≥ 30) based on the World Health Organization (WHO)’s definition [16]. Based on the location of the study sites, participants were categorized into two different regions: Europe and the United States of America (US).

### 2.3. Statistical Analyses

The outcomes of this study were intake frequency status (infrequent, light/moderate, and heavy) of total alcohol and three alcoholic beverage types (beer, wine, and spirits), and the primary predictors were PCa risk status (PCa vs. non-PCa) and PCa aggressiveness status (yes/no). The participants’ PCa case/aggressiveness status, age, BMI, smoking status, and region of study sites by alcohol intake patterns were summarized using descriptive statistics. The alcohol intake pattern agreement between each beverage type with total alcohol status was tested using the Kappa coefficient. The high Kappa coefficient indicates a higher agreement between the two measures. The associations between PCa risk/aggressiveness status and other selected factors associated with total and specific-beverage alcohol intake were tested using the chi-square test for categorical variables and the analysis of variance (ANOVA) test for continuous variables. We assessed associations of PCa case/aggressiveness status with alcohol intake patterns adjusting for age, BMI, smoking status, and region using multinomial logistic regression. The *p*-values, odds ratios (ORs), and their 95% confidence intervals (CIs) were reported for each model. In addition, forest plots of adjusted ORs of light/moderate and heavy intake for PCa risk and aggressiveness status were presented. All *p*-values were based on two-sided tests.

## 3. Results

The participants’ demographic and related characteristics are shown in Table 1 and Table S2. We evaluated 10,029 men with European ancestry, including 4676 non-PCa men and 5353 PCa patients. Among PCa patients, there were 4507 (84.3%) patients with non-aggressive PCa and 837 (15.7%) with aggressive PCa. For total alcohol intake, most men (64.3%) had a light to moderate level of total alcohol intake, and 14.3% of men were heavy drinkers. For specific beverage types, heavy intake prevalence was 8.8%, 4.7%, and 2.2% for intake of beer, wine, and spirits, respectively. We tested agreement between total alcohol intake with each of the three beverage types using the Kappa coefficient. The agreement between total alcohol and beer intake (Kappa = 0.66) was higher than wine (Kappa = 0.53) and spirits (Kappa = 0.31). Thus, the total alcohol status was primarily driven by beer intake.

**Figure 1:**
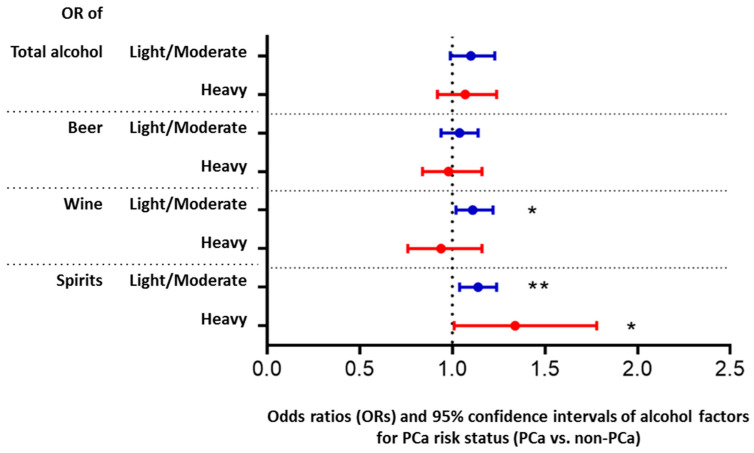
Impact of prostate cancer (PCa) risk status on intake of total alcohol and specific alcoholic beverage types. The results are based on multinomial logistic model with an alcohol factor (three sub-groups: infrequent, light/moderate, and heavy intake) as the outcome. Factors in the model were PCa risk status (PCa/ non-PCa), age, BMI, smoking, and region. Odds ratio (OR) and 95% confidence interval of light/moderate (vs. infrequent intake) and heavy (vs. infrequent intake) for PCa vs. non-PCa were shown. *: *p* < 0.05, **: *p <* 0.01.

The PCa status (PCa/non-PCa), age, BMI status, smoking, and region of study site were significantly associated with intake of total alcohol and three beverage types (beer, wine, and spirits) with all *p*-values < 0.001. Age distribution was inversely associated with total alcohol intake (*p* < 0.001). As shown in Table 1, light/moderate and heavy drinkers of total alcohol were younger than infrequent drinkers. The mean age for infrequent, light/moderate, and heavy drinkers of total alcohol were 64.4, 63.2, 63.8, respectively. Similarly, heavy beer drinkers were younger than infrequent drinkers (62.7 vs. 64.7, *p* < 0.001). However, heavy wine drinkers and heavy spirits drinkers tended to be older (mean age = 66.0 and 65.1, respectively) than infrequent drinkers. These age effects remained significant after adjusting for other factors. As shown in Figure 1 and Table S2, men with obesity were less likely to have heavy total intake (adjusted OR = 0.73, *p* = 0.005) and heavy beer intake (adjusted OR = 0.59, *p* < 0.001), but had a similar heavy intake of wine and spirits compared with men who were normal/underweight, adjusting for other factors. In addition, men with smoking experience (former or current smoking) tended to report higher alcohol use. This trend applied to all alcoholic beverage types, except heavy wine users. Men living in the US tended to drink less total alcohol than men residing in Europe (adjusted OR = 0.10 for light/moderate intake, and OR = 0.06 for heavy intake of total alcohol use for US vs. European). However, US men more often reported heavy intake of spirits than European men (adjusted OR = 3.2, *p* < 0.0001).

The association of PCa risk status (PCa vs. non-PCa) with alcohol intake without adjusting other factors is shown in Table 1. PCa risk status was significantly associated with intake of total alcohol and three alcoholic beverage types (*p* < 0.001). Prevalence of heavy intake of total alcohol was significantly different for non-PCa men, patients with non-aggressive PCa, and patients with aggressive PCa (Table 1 and Table 2, 12.9%, 14.9%, and 18.7%, respectively; *p* < 0.001). PCa patients had higher prevalence of current heavy alcohol intake than non-PCa subjects (15.5% vs. 12.9%, crude OR of heavy intake = 1.71, *p <* 0.001). For beer intake, PCa patients had significantly higher prevalence of current light/moderate (58.6% vs. 52.4%, crude OR = 1.39, *p <* 0.001) and heavy beer intake (9.6% vs. 8.0%, crude OR = 1.50, *p <* 0.001) compared to non-PCa men. Wine intake showed a similar pattern as beer intake for PCa patients, but PCa patients were not significantly associated with heavy spirits intake (*p* = 0.614). PCa patients had higher prevalence of current light/moderate spirits intake (44.6% vs. 38.9%, crude OR = 1.27, *p <* 0.001), but had the same heavy spirit intake prevalence (2.2% vs. 2.2%) compared with the non-PCa men.

The associations of the PCa risk status with alcohol intake after adjusting all the selected variables (age, obesity status, smoking, and region) are shown in Figure 1 and Table S2. After adjusting other factors, the association between total alcohol and PCa risk status became insignificant. However, PCa patients had significantly different intake patterns in some alcoholic beverage types even after adjusting for confounding factors. PCa patients were more likely to be light/moderate wine drinkers than non-PCa men (adjusted OR = 1.11, *p* = 0.018), while PCa status was not significantly associated with heavy wine intake (*p =* 0.554). For spirits intake, PCa patients were more likely to be light/moderate spirits drinkers (adjusted OR = 1.14, *p =* 0.003) and heavy spirits drinkers (adjusted OR = 1.34, *p =* 0.040) compared to non-PCa men. PCa risk status was not significantly associated with total alcohol intake (light/moderate: *p* = 0.077, heavy: *p* = 0.414) and beer intake (light/moderate: *p* = 0.484, heavy: *p* = 0.850).

**Figure 2:**
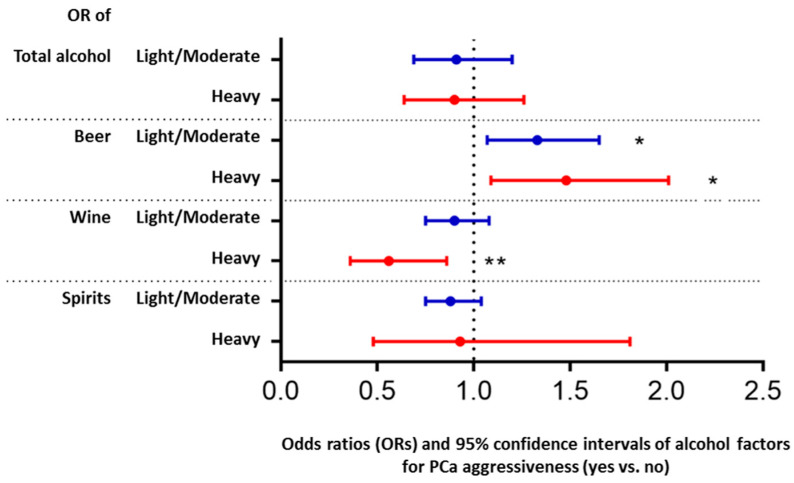
Impact of prostate cancer (PCa) aggressiveness on intake of total alcohol and specific alcoholic beverage types. The results are based on a multinomial logistic model with an alcohol factor (three sub-groups: infrequent, light/moderate, and heavy intake) as the outcome. Factors in the model were PCa aggressiveness status (yes/no), age, BMI, smoking, and region. Odds ratio (OR) and 95% confidence interval of light/moderate (vs. infrequent intake) and heavy (vs. infrequent intake) for PCa vs. non-PCa were shown. *: *p* < 0.05, **: *p <* 0.01.

For PCa patients, PCa aggressiveness status was only significantly associated with patterns of total alcohol intake (*p <* 0.001) and beer intake (*p* < 0.001), but not for wine (*p* = 0.117) and spirits intake (*p* = 0.196) based on the unadjusted results shown in Table 2. As shown in Table 2 and Table S3, patients with aggressive PCa tended to have a heavy intake of total alcohol than patients with non-aggressive PCa (18.7% vs. 14.9%, crude OR = 2.15, *p* < 0.001). In addition, patients with aggressive PCa had higher prevalence of light/moderate beer intake (67.0% vs. 57.1%, crude OR of light/moderate intake = 1.99, *p* < 0.001) and higher prevalence of heavy beer intake (13.0% vs. 8.9%, crude OR = 2.47, *p* < 0.001) compared to patients with non-aggressive PCa. The adjusting associations between PCa aggressiveness and alcohol intake are shown in Figure 2 and Table S3. Although PCa aggressiveness was not significantly associated with total alcohol intake (*p* = 0.503 for light/moderate and *p* = 0.530 for heavy), PCa aggressiveness was significantly associated with intake of beer (light/moderate and heavy intake) and wine (heavy intake). Patients with aggressive PCa were more likely to be light/moderate and heavy beer drinkers than patients with non-aggressive PCa (adjusted OR of light/moderate = 1.33, *p* = 0.012; adjusted OR of heavy = 1.48, *p* = 0.013). However, patients with aggressive PCa were less likely to be heavy wine drinkers than patients with non-aggressive PCa (adjusted OR of heavy = 0.56, *p* = 0.009).

Among PCa patients, the associations of obesity, smoking, and region with alcohol intake varied by alcoholic beverage types (Table S3). In general, men with overweight and obesity had less total alcohol and beer intake based on both unadjusted and adjusted results. However, obese PCa patients tended to be heavy wine drinkers (adjusted OR = 1.79, *p* = 0.012), but BMI was not associated with patterns of spirits intake after adjusting for other factors. For smoking status, PCa patients with former or current smoking were likely to drink more total alcohol, beer, and spirits compared to patients without smoking, but the smoking status was not associated with heavy wine intake based on adjusted results. For the region of study sites, US PCa patients drank less total alcohol, beer, and wine based on both unadjusted and adjusted results. US PCa patients had a higher chance of being heavy spirits drinkers (adjusted OR = 4.10, *p* < 0.001) than patients living in Europe based on the adjusted results.

## 4. Discussion

Our study findings showed that the intake pattern of total alcohol was similar for non-PCa men and PCa patients after adjusting for other factors. However, the intake patterns for some specific alcoholic beverages were different by the PCa case and aggressiveness status. PCa patients were likely to drink more wine (light/moderate) and spirits (light/moderate and heavy) than non-PCa men, but the beer intake frequency was similar between PCa patients and non-PCa men. Among PCa patients, patients with aggressive PCa tended to drink more beer (light/moderate and heavy) but were less likely to be heavy wine drinkers than patients with non-aggressive PCa.

A growing number of studies showed that excessive beer intake is associated with a worse PCa prognosis [3,17], but wine may have a potential protective effect on PCa [17,18]. Our results are consistent with previous studies, although alcohol intake information collected after a cancer diagnosis cannot be used for causal inference. Our study showed that patients with aggressive PCa tended to drink more beer (light/moderate and heavy intake) and less wine than patients with non-aggressive PCa. The prevalence of heavy beer intake was 8%, 8.9%, and 13% for non-PCa men, patients with non-aggressive PCa, and aggressive PCa. Based on meta-analysis analyses with only longitudinal cohort studies, the impact of alcohol on PCa clinical outcomes (non-aggressive PCa and aggressive PCa vs. non-PCa) varied by alcoholic beverage types (beer, wine, and spirits), and these associations are not linear. However, the total alcohol intake was not significantly associated with both types of PCa [4]. The same meta-analysis study observed that the intake of beer and spirits was associated with an increased risk of non-aggressive PCa, and intake of wine and spirits was associated with a high risk of aggressive PCa [4]. Another study reported that heavy beer intake (≥5 days per week) was associated with a higher risk of PCa aggressiveness (OR = 1.66, *p* = 0.012), but wine intake was associated with reduced PCa aggressiveness, and no significant association was found for spirits [17]. Moreover, a large longitudinal study, which followed ~47,000 cancer-free men for 27 years, reported that total alcohol intake for PCa patients was not associated with lethal PCa, but moderate red wine intake was associated with a lower risk of lethal PCa [18].

Most alcohol studies reported total alcohol patterns without considering the types of alcoholic beverages. Among these beverage types, spirits have a higher ethanol concentration than beer and wine [19]. In this study, beer drinking was more prevalent than the drinking of wine and spirits, so the measure of total alcohol intake was primarily driven by beer intake. This observation was consistent with results from a previous study [3]. Given the partly opposite associations seen for different alcoholic beverage types, usage of total alcohol intake without considering beverage types is challenging to reveal the true relationship between alcohol intake and PCa status. In addition to the association of alcohol intake with PCa, we also evaluated the associations of age and smoking with alcohol intake. Our results are consistent with other studies in that young men and current smokers tended to have excessive alcohol drinking. These associations were similar for the general population and cancer survivors [12,20].

Our study findings suggested that alcohol intake behavior for PCa patients can be improved. Because of cancer survivors’ sub-optimal health history, cancer survivors are likely to have a stronger motivation to change their lifestyle and adhere to a healthy lifestyle than the general population. It has been suggested that cancer diagnosis has been offered as an excellent educational moment for patients to improve their health behaviors [21]. In addition to the potentially harmful impact of excessive alcohol intake on PCa risk and aggressiveness, heavy alcohol consumption is also associated with many chronic diseases that PCa patients may have [22]. PCa is considered a chronic disease with a five-year relative survival rate of 98% for PCa patients overall [23]. Due to the older age of PCa patients, many of them had multiple comorbid conditions, such as diabetes, hypertension, and renal disease [24,25]. With many harmful impacts of heavy alcohol intake, we would expect that PCa survivors tend to reduce alcohol intake more than the general population. However, our results showed that the prevalence of heavy alcohol intake for all alcoholic beverages (beer, wine, and spirits) for PCa patients was either similar or higher than non-PCa men. Our findings are consistent with our previous population-based study on a large-scale US population survey study during 2012–2017. In this population-based study, the prevalence of heavy alcohol intake was similar for individuals regardless of their cancer status (yes/no), type of cancer (alcohol-related cancer or not), and length of cancer history [12]. In addition, a large-scale longitudinal cohort study compared alcohol intake status between pre-PCa vs. post-PCa diagnosis and observed that the majority of men (61%) remained in the same alcohol drinking category after PCa diagnosis, and only 19% PCa patients had decreased alcohol intake [18]. It has been shown that alcohol intake patterns and preference of beverage types are affected by many factors, such as socio-demographics, psychological conditions, social and cultural norms, taste perception, and genetics [26,27]. By understanding the intake of alcoholic beverage types for PCa patients, custom alcohol intervention by beverage types can be developed and promoted.

The strengths of this study include data from a large sample size of participants from the US and Europe and a thorough evaluation of light/moderate and heavy alcohol intake. However, there are some limitations of this study. First, there is potential imperfect reporting of alcohol intake, either by imperfect recall or willful misreporting. Second, this study may have population heterogeneity because of participants from multiple sites in the US and different countries in Europe. Third, alcohol intake status for PCa patients is based on post-diagnosis alcohol behavior data, so the results cannot be used for inferring a causal relationship between alcohol intake and PCa status. Finally, this study focuses only on men with European ancestry, so the results may not be applied to other races.

## 5. Conclusions

In summary, this study compared the alcohol intake patterns of different beverage types by PCa risk and aggressiveness status. PCa patients had different drinking patterns in specific beverage types, although PCa patients had a similar total alcohol intake pattern compared with non-cancer men. We report detailed and valuable information on the intake patterns of beer, wine, and spirits by PCa risk and aggressiveness status. This information can be a solid reference for developing precision alcohol prevention intervention for PCa patients.

## Figures and Tables

**Table 1:** Demographic and related characteristics by alcohol intake status for study participants (prostate cancer [PCa] patients + non-PCa men, N = 10,029).

	Total Alcohol Intake ^1^			Beer Intake ^1^			Wine Intake ^1^			Spirits Intake ^1^		
	Infrequent	Light/ Moderate	Heavy	Infrequent	Light/ Moderate	Heavy	Infrequent	Light/ Moderate	Heavy	Infrequent	Light/ Moderate	Heavy
	N (%)	N (%)	N (%)	N (%)	N (%)	N (%)	N (%)	N (%)	N (%)	N (%)	N (%)	N (%)
Total	2016 (21.4)	6056 (64.3)	1343 (14.3)	3513 (35.5)	5520 (55.7)	872 (8.8)	3889 (40.4)	5278 (54.9)	456 (4.7)	5415 (55.8)	4067 (42.0)	215 (2.2)
**Prostate cancer (PCa)**												
No Yes	1124 (25.4) 892 (17.9)	2728 (61.7) 3328 (66.6)	569 (12.9) 774 (15.5) ***	1829 (39.6) 1684 (31.8)	2419 (52.4) 3101 (58.6)	367 (8.0) 505 (9.6) ***	2009 (44.4) 1880 (36.9)	2329 (51.5) 2949 (57.9)	188 (4.1) 268 (5.2) ***	2664 (58.9) 2751 (53.2)	1760 (38.9) 2307 (44.6)	102 (2.2) 113 (2.2) ***
**Age** (mean ± SD) ^2^	64.4 ± 6.2	63.2 ± 7.0	63.8 ± 7.2 ***	64.7 ± 6. 5	63.0 ± 7.0	62.7 ± 7.2 ***	63.9 ± 6.7	63.2 ± 7.0	66.0 ± 7.0 ***	63.8 ± 6.8	63.3 ± 7.1	65.1 ± 6.6 ***
**BMI status** ^3^												
Normal/Underweight	460 (17.3)	1798 (67.7)	398 (15.0)	816 (28.9)	1692 (59.9)	316 (11.2)	972 (35.6)	1650 (60.4)	110 (4.0)	1551 (56.6)	1151 (42.0)	39 (1.4)
Overweight	929 (20.0)	3026 (65.1)	692 (14.9)	1717 (35.3)	2732 (56.2)	410 (8.4)	1833 (38.6)	2659 (56.1)	252 (5.3)	2597 (54.3)	2062 (43.1)	122 (2.6)
Obese	608 (31.0)	1125 (57.3)	229 (11.7) ***	943 (46.2)	974 (47.8)	123 (6.0) ***	1010 (50.8)	889 (44.7)	91 (4.6) ***	1173 (58.3)	787 (39.1)	53 (2.6) ***
**Smoking**												
Never	896 (24.4)	2391 (65.2)	382 (10.4)	1467 (38.1)	2152 (55.9)	228 (5.9)	1565 (41.7)	2042 (54.5)	142 (3.8)	2353 (62.7)	1345 (35.9)	53 (1.4)
Former	970 (20.9)	2933 (63.2)	740 (15.9)	1745 (36.0)	2628 (54.2)	474 (9.8)	1880 (39.7)	2616 (55.2)	240 (5.1)	2540 (53.1)	2110 (44.1)	134 (2.8)
Current	145 (13.7)	697 (65.8)	217 (20.5)***	290 (25.0)	704 (60.8)	164 (14.2) ***	428 (39.3)	590 (54.1)	72 (6.6) ***	492 (44.1)	596 (53.4)	28 (2.5) ***
**Region**												
Europe	175 (4.4)	2930 (74.4)	833 (21.2)	613 (14.0)	3125 (71.5)	635 (14.5)	1089 (26.6)	2702 (65.9)	310 (7.5)	1940 (46.6)	2187 (52.6)	35 (0.8)
USA	1841 (33.6)	3126 (57.1)	510 (9.3) ***	2900 (52.4)	2395 (43.3)	237 (4.3) ***	2800 (50.7)	2576 (46.7)	146 (2.6) ***	3475 (62.8)	1880 (34.0)	180 (3.2) ***

^1^ *p*-values for categorical variables were based on chi-square test, *p*-values for continuous variables were based on ANOVA test; ***: *p <* 0.001. ^2^ SD: standard deviation. ^3^ Normal/underweight (body mass index (BMI) < 25), overweight (BMI: 25–29.9), and obesity (BMI ≥ 30).

**Table 2:** Demographic and related characteristics by alcohol intake status for prostate cancer (PCa) patients (N = 5344).

	Total Alcohol Intake ^1^			Beer Intake ^1^			Wine Intake ^1^			Spirits Intake ^1^		
	Infrequent	Light/ Moderate	Heavy	Infrequent	Light/ Moderate	Heavy	Infrequent	Light/ Moderate	Heavy	Infrequent	Light/ Moderate	Heavy
	N (%)	N (%)	N (%)	N (%)	N (%)	N (%)	N (%)	N (%)	N (%)	N (%)	N (%)	N (%)
Total	892 (17.9)	3321 (66.6)	772 (15.5)	1682 (31.9)	3095 (58.6)	504 (9.5)	1879 (36.9)	2942 (57.8)	267 (5.3)	2746 (53.2)	2303 (44.6)	113 (2.2)
**PCa aggressiveness**												
No Yes	810 (19.1) 82 (11.1)	2802 (66.0) 519 (70.2)	634 (14.9) 138 (18.7) ***	1518 (34.0) 164 (20.0)	2547 (57.1) 548 (67.0)	398 (8.9) 106 (13.0) ***	1621 (37.5) 258 (33.6)	2477 (57.3) 465 (60.6)	223 (5.2) 44 (5.7)	2334 (53.3) 412 (52.4)	1940 (44.3) 363 (46.2)	102 (2.3) 11 (1.4)
**Age** (mean ± SD) ^2^	64.2± 6.0	63.1± 7.1	63.6± 7.3 ***	64.8± 6.4	62.9± 7.2	62.7± 7.3 ***	63.7± 6.7	63.0± 7.1	66.0± 7.2 ***	63.8± 7.0	63.0± 7.1	64.4± 6.4 ***
**BMI status** ^3^												
Normal/Underweight	206 (13.4)	1094 (71.0)	241 (15.6)	409 (24.7)	1050 (63.4)	197 (11.9)	530 (33.4)	999 (63.0)	57 (3.6)	860 (53.7)	713 (44.5)	28 (1.8)
Overweight	432 (17.7)	1619 (66.4)	386 (15.8)	850 (33.2)	1480 (57.9)	228 (8.9)	883 (35.6)	1447 (58.4)	148 (6.0)	1321 (52.4)	1139 (45.2)	59 (2.3)
Obese	250 (27.4)	533 (58.5)	128 (14.1) ***	409 (43.1)	479 (50.4)	62 (6.5) ***	424 (45.9)	439 (47.6)	60 (6.5) ***	511 (54.4)	402 (42.8)	26 (2.8)
**Smoking**												
Never	410 (20.4)	1367 (68.0)	233 (11.6)	720 (33.8)	1275 (59.8)	137 (6.4)	777 (37.8)	1191 (57.9)	89 (4.3)	1250 (60.4)	792 (38.2)	29 (1.4)
Former	416 (17.7)	1520 (64.7)	412 (17.6)	816 (33.2)	1379 (56.1)	262 (10.7)	882 (36.9)	1368 (57.3)	138 (5.8)	1220 (50.2)	1141 (47.0)	68 (2.8)
Current	65 (10.8)	410 (68.3)	125 (20.8) ***	139 (21.1)	418 (63.5)	101 (15.4) ***	213 (34.6)	363 (59.0)	39 (6.3)	259 (40.9)	358 (56.6)	16 (2.5) ***
**Region**												
Europe	105 (4.1)	1909 (74.7)	541 (21.2)	399 (14.1)	2014 (71.4)	409 (14.5)	692 (26.2)	1752 (66.4)	196 (7.4)	1261 (46.7)	1418 (52.5)	22 (0.8)
USA	787 (32.4)	1412 (58.1)	231 (9.5) ***	1283 (52.2)	1081 (44.0)	95 (3.9) ***	1187 (48.5)	1190 (48.6)	71 (2.9) ***	1485 (60.3)	885 (36.0)	91 (3.7) ***

^1^ *p*-values for categorical variables were based on chi-square test, *p*-values for continuous variables were based on ANOVA test; ***: *p <* 0.001. ^2^ SD: standard deviation. ^3^ Normal/underweight (body mass index (BMI) < 25), overweight (BMI: 25–29.9), and obesity (BMI ≥ 30).

## Data Availability

The data used in this project are available from the Prostate Cancer Association Group to Investigate Cancer Associated Alterations in the Genome consortium (PRACTICAL Consortium, http://practical.icr.ac.uk/blog/?page_id=1244, accessed on 30 January 2018), but restrictions apply to the availability of these data.

## Supplementary Materials

The following supporting information can be downloaded at https://www.mdpi.com/article/10.3390/cancers14081981/s1, Figure S1. Flow chart of study population, Table S1: Summary of prostate cancer (PCa) status and related information by study sites, Table S2: Prostate cancer risk status and other selected factors associated with intake of total alcohol and specific alcoholic beverage types, Table S3: Prostate cancer (PCa) aggressiveness status and other selected factors associated with intake of total alcohol and specific alcoholic beverage types for PCa patients.
