# Supplementary material for: Intake Patterns of Specific Alcoholic Beverages by Prostate Cancer Status
Source: Cancers (Basel). 2022 Apr 14;14(8):1981. doi: 10.3390/cancers14081981 (PMC9024489; doi:10.3390/cancers14081981)
Supplement: Supplementary file 1 [file cancers-14-01981-s001.zip › cancers-1646195-supplementary.pdf]

Supplementary Materials

Figure S1. Flow chart of the study population

84,239 men with valid prostate cancer status and European ancestry

↓ With current alcohol intake data

11,493

↓ With valid alcoholic beverage information

10,029

Table S1. Summary of prostate cancer (PCa) status and related information by study sites

Study site	Method of collecting alcohol data	Country	Sample size	Non-PCa N (%)		PCa N (%)	
COSM	questionnaire	Sweden[1]	3146	1028	(32.7)	2118	(67.3)
ESTHER	questionnaire	Germany[1]	385	197	(51.2)	188	(48.8)
Hamburg-Zagreb	Interview	Croatia[1]	6	0	(0)	6	(100)
KULEUVEN	Interview	Belgium[1]	6	2	(33.3)	4	(66.7)
MCC-Spain	Questionnaire	Spain[1]	833	359	(43.1)	474	(56.9)
PRAGGA	Questionnaire	Spain[1]	93	0	(0)	93	(100)
SWOG-PCPT	Questionnaire	USA	2045	1045	(51.1)	100	(48.9)
SWOG-SELECT	Questionnaire	USA	3515	2045	(58.2)	1470	(41.8)
Total	-	-	10029	4676	(46.6)	3553	(35.4)

[1]In European

Table S2. Prostate cancer risk status and other selected factors associated with intake of total alcohol and specific alcoholic beverage types

	Total alcohol				Beer intake			
	Univariate Model		Multivariable Model[1] (N=9135)		Univariate Model		Multivariable Model[1] (N=9585)	
Factor	Light/moderate	Heavy	Light/moderate	Heavy	Light/moderate	Heavy	Light/moderate	Heavy
Prostate cancer								
No	1	1	1	1	1	1	1	1
Yes	1.54 (1.39, 1.70)***	1.71 (1.49, 1.97)***	1.10 (0.99, 1.23)	1.07 (0.92, 1.24)	1.39 (1.28, 1.52)***	1.50 (1.29, 1.74)***	1.04 (0.94, 1.14)	0.98 (0.84, 1.16)
Age	0.98 (0.97, 0.98)***	0.99 (0.98, 1.00)*	0.98 (0.97, 0.98)***	0.99 (0.98, 1.00)*	0.97 (0.96, 0.97)***	0.96 (0.95, 0.97)***	0.96 (0.95, 0.96)***	0.95 (0.94, 0.96)***
BMI status[2]								
Normal/Underweight	1	1	1	1	1	1	1	1
Overweight	0.83 (0.74, 0.95)**	0.86 (0.73, 1.02)	1.07 (0.93, 1.23)	1.14 (0.95, 1.36)	0.77 (0.69, 0.85)***	0.62 (0.52, 0.73)***	0.96 (0.86, 1.08)	0.81 (0.67, 0.97)*
Obese	0.47 (0.41, 0.55)***	0.44 (0.36, 0.53)***	0.74 (0.63, 0.86)**	0.73 (0.58, 0.91)**	0.50 (0.44, 0.56)***	0.34 (0.27, 0.42)***	0.76 (0.66, 0.87)***	0.59 (0.46, 0.76)***
Smoking								
Never	1	1	1	1	1	1	1	1
Former	1.13 (1.02, 1.26)*	1.79 (1.54, 2.09)***	1.24 (1.11, 1.39)**	2.01 (1.70, 2.37)***	1.03 (0.94, 1.12)	1.75 (1.47, 2.08)***	1.14 (1.03, 1.26)*	2.06 (1.71, 2.48)***
Current	1.80 (1.48, 2.19)***	3.51 (2.76, 4.47)***	0.92 (0.74, 1.14)	1.63 (1.25, 2.14)**	1.66 (1.42, 1.93)***	3.64 (2.87, 4.61)***	0.84 (0.71, 1.00)	1.60 (1.23, 2.07)***
Region								
Europe	1	1	1	1	1	1	1	1
USA	0.10 (0.09, 0.12)***	0.06 (0.05, 0.07)***	0.10 (0.09, 0.12)***	0.06 (0.05, 0.08)***	0.16 (0.15, 0.18)***	0.08 (0.07, 0.09)***	0.15 (0.13, 0.16)***	0.07 (0.06, 0.09)***

	Wine intake				Spirits intake			
	Univariate Model		Multivariable Model[1] (N= 9332)		Univariate Model		Multivariable Model[1] (N=9403)	
Factor	Light/moderate	Heavy	Light/moderate	Heavy	Light/moderate	Heavy	Light/moderate	Heavy
Prostate cancer								
No	1	1	1	1	1	1	1	1
Yes	1.35 (1.25, 1.47)***	1.52 (1.25, 1.85)***	1.11 (1.02, 1.22)*	0.94 (0.76, 1.16)	1.27 (1.17, 1.38)***	1.07 (0.82, 1.41)	1.14 (1.04, 1.24)**	1.34 (1.01, 1.78)*
Age	0.99 (0.98, 0.99)***	1.05 (1.03, 1.06)***	0.99 (0.98, 0.99)***	1.04 (1.03, 1.06)***	0.99 (0.98, 1.00)***	1.03 (1.01, 1.05)**	0.99 (0.99, 1.00)*	1.03 (1.01, 1.06)**
BMI status[2]								
Normal/Underweight	1	1	1	1	1	1	1	1
Overweight	0.85 (0.77, 0.94)**	1.22 (0.96, 1.54)	0.97 (0.88, 1.08)	1.46 (1.13, 1.89)**	1.07 (0.97, 1.18)	1.87 (1.30, 2.69)***	1.18 (1.07, 1.30)**	1.59 (1.10, 2.30)*
Obese	0.52 (0.46, 0.59)***	0.80 (0.60, 1.07)	0.67 (0.59, 0.76)***	1.13 (0.81, 1.56)	0.90 (0.80, 1.02)	1.80 (1.18, 2.74)**	1.15 (1.01, 1.30)*	1.42 (0.92, 2.19)
Smoking								
Never	1	1	1	1	1	1	1	1
Former	1.07 (0.98, 1.17)	1.41 (1.13, 1.75)**	1.13 (1.03, 1.24)*	1.40 (1.10, 1.78)**	1.45 (1.33, 1.59)***	2.34 (1.70, 3.23)***	1.47 (1.34, 1.61)***	2.24 (1.61, 3.10)***
Current	1.06 (0.92, 1.22)	1.85 (1.37, 2.51)***	0.72 (0.62, 0.84)***	1.27 (0.91, 1.77)	2.12 (1.85, 2.43)***	2.53 (1.58, 4.04)***	1.65 (1.43, 1.91)***	3.54 (2.16, 5.80)***
Region								
Europe	1	1	1	1	1	1	1	1
USA	0.37 (0.34, 0.41)***	0.18 (0.15, 0.23)***	0.38 (0.34, 0.41)***	0.21 (0.17, 0.27)***	0.48 (0.44, 0.52)***	2.87 (1.99, 4.14)***	0.48 (0.44, 0.52)***	3.20 (2.15, 4.77)***

[1]Based on multinomial logistic models with an alcohol factor as the outcome adjusting all factors in this table; *: p<0.05, **: P<0.01, ***: P<0.001

[2]Normal/underweight (body mass index (BMI)<25), overweight (BMI: 25-29.9), and obesity (BMI≥30)

Table S3. Prostate cancer (PCa) aggressiveness status and other selected factors associated with intake of total alcohol and specific alcoholic beverage types for PCa patients

	Total alcohol intake				Beer intake			
Factor	Univariate Model		Multivariable Model[1] (N=4777)		Univariate Model		Multivariable Model[1] (n=5045)	
	Light/moderate	Heavy	Light/moderate	Heavy	Light/moderate	Heavy	Light/moderate	Heavy
PCa aggressiveness								
No	1	1	1	1	1	1	1	1
Yes	1.83 (1.43, 2.34)***	2.15 (1.61, 2.88)***	0.91 (0.69, 1.20)	0.90 (0.64, 1.26)	1.99 (1.65, 2.40)***	2.47 (1.89, 3.22)***	1.33 (1.07, 1.65)*	1.48 (1.09, 2.01)*
Age	0.98 (0.97, 0.99)***	0.99 (0.97, 1.00)	0.98 (0.97, 0.99)***	0.99 (0.97, 1.00)	0.96 (0.95, 0.97)***	0.96 (0.94, 0.97)***	0.95 (0.94, 0.96)***	0.94 (0.93, 0.96)***
BMI status[2]								
Normal/Underweight	1	1	1	1	1	1	1	1
Overweight	0.71 (0.59, 0.85)*	0.76 (0.61, 0.96)***	0.92 (0.76, 1.12)	1.00 (0.78, 1.29)	0.68 (0.59, 0.78)***	0.56 (0.45, 0.70)***	0.85 (0.72, 1.00)*	0.72 (0.56, 0.92)**
Obese	0.40 (0.33, 0.50)***	0.44 (0.33, 0.58)***	0.66 (0.52, 0.83)***	0.71 (0.52, 0.98)*	0.46 (0.38, 0.54)***	0.31 (0.23, 0.43)***	0.71 (0.59, 0.87)***	0.56 (0.40, 0.80)**
Smoking								
Never	1	1	1	1	1	1	1	1
Former	1.10 (0.94, 1.28)	1.74 (1.41, 2.15)***	1.19 (1.01, 1.41)*	1.95 (1.55, 2.46)***	0.95 (0.84, 1.08)	1.69 (1.34, 2.12)***	1.05 (0.91, 1.21)	1.97 (1.54, 2.54)***
Current	1.89 (1.42, 2.51)***	3.38 (2.41, 4.76)***	0.87 (0.63, 1.19)	1.47 (1.00, 2.14)*	1.70 (1.37, 2.10)***	3.82 (2.79, 5.23)***	0.82 (0.64,1.04)	1.59 (1.12, 2.26)**
Region								
Europe	1	1	1	1	1	1	1	1
USA	0.10 (0.08, 0.12)***	0.06 (0.04, 0.07)***	0.10 (0.08, 0.13)***	0.06 (0.05, 0.08)***	0.17 (0.15, 0.19)***	0.07 (0.06, 0.09)***	0.15 (0.13-0.17)***	0.07 (0.05, 0.09)***

	Wine intake				Spirits intake			
Factor	Univariate Model		Multivariable Model[1] (n=4874)		Univariate Model		Multivariable Model[1] (n=4945)	
	Light/moderate	Heavy	Light/moderate	Heavy	Light/moderate	Heavy	Light/moderate	Heavy
PCa aggressiveness								
No	1	1	1	1	1	1	1	1
Yes	1.18 (1.00, 1.39)*	1.24 (0.87, 1.76)	0.90 (0.75, 1.08)	0.56 (0.36, 0.86)**	1.06 (0.91, 1.24)	0.61 (0.33, 1.15)	0.88 (0.75, 1.04)	0.93 (0.48, 1.81)
Age	0.99 (0.98, 0.99)**	1.05 (1.03, 1.07)***	0.99 (0.98, 1.00)*	1.05 (1.03, 1.08)***	0.99 (0.98, 0.99)***	1.01 (0.99, 1.04)	0.98 (0.98, 1.00)**	1.02 (0.99, 1.05)
BMI status[2]								
Normal/Underweight	1	1	1	1	1	1	1	1
Overweight	0.87 (0.76, 1.00)*	1.56 (1.13, 2.16)**	1.00 (0.87, 1.15)	1.96 (1.35, 2.83)***	1.04 (0.92, 1.18)	1.37 (0.87, 2.17)	1.14 (1.00, 1.30)	1.10 (0.69, 1.75)
Obese	0.55 (0.46, 0.65)***	1.32 (0.90, 1.93)	0.71 (0.59, 0.85)***	1.79 (1.14, 2.81)*	0.95 (0.81, 1.12)	1.56 (0.91, 2.69)	1.19 (1.00, 1.41)	1.10 (0.63, 1.93)
Smoking								
Never	1	1	1	1	1	1	1	1
Former	1.01 (0.89, 1.15)	1.37 (1.03, 1.81)*	1.05 (0.92, 1.19)	1.33 (0.96, 1.84)	1.48 (1.31, 1.67)***	2.40 (1.54, 3.74)***	1.48 (1.31, 1.67)***	2.35 (1.50, 3.69)***
Current	1.11 (0.92, 1.35)	1.60 (1.07, 2.40)*	0.79 (0.64, 0.96)*	1.23 (0.77,1.95)	2.18 (1.82, 2.62)***	2.66 (1.43, 4.97)**	1.72 (1.42, 2.09)***	4.13 (2.13, 8.02)***
Region								
Europe	1	1	1	1	1	1	1	1
USA	0.40 (0.35, 0.45)***	0.21 (0.16, 0.28)***	0.39 (0.34, 0.45)***	0.24 (0.17, 0.34)***	0.53 (0.47, 0.59)***	3.51 (2.19, 5.63)***	0.50 (0.44, 0.57)***	4.10 (2.40, 7.00)***

[1]Based on multinomial logistic models with an alcohol factor as the outcome adjusting all factors in this table; *: p<0.05, **: P<0.01, ***: P<0.001
[2]Normal/underweight (body mass index (BMI)<25), overweight (BMI: 25-29.9), and obesity (BMI≥30)

Acknowledgments (in addition to the main Acknowledgments section)

We would like to thank the support from the following researchers from the PRACTICAL Consortium:

Ian M. Thompson Jr. [1], Gemma Castaño-Vinyals [2,3,4,5], Bernd Holleczek [6], Hannah Stocker [7], Maria Elena Martinez [8], Tomislav Kulis [9], Rosalind A. Eeles [10,11], Zsofia Kote-Jarai [10], Christopher A. Haiman [12], Kenneth R. Muir [13], Janet L. Stanford [14,15]

[1] Christus Santa Rosa Hospital – Medical Center, San Antonio, Texas 78229, USA

[2] Universitat Pompeu Fabra (UPF), Barcelona, Spain

[3] CIBER Epidemiología y Salud Pública (CIBERESP), 28029 Madrid, Spain

[4] University of Cantabria, 39005 Santander, Spain

[5] CIBER Epidemiología y Salud Pública (CIBERESP), 28029 Madrid, Spain

[6] Saarland Cancer Registry, 66119 Saarbrücken, Germany

[7] Division of Preventive Oncology, German Cancer Research Center (DKFZ) and National Center for Tumor Diseases (NCT), Im Neuenheimer Feld 460, 69120 Heidelberg, Germany

[8] University of California San Diego, Moores Cancer Center, Department of Family Medicine and Public Health, University of California San Diego, La Jolla, CA 92093-0012, USA

[9] Department of Urology, University Hospital Center Zagreb, University of Zagreb School of Medicine, Zagreb, Croatia

[10] The Institute of Cancer Research, London, SM2 5NG, UK

[11] Royal Marsden NHS Foundation Trust, London, SW3 6JJ, UK

[12] Center for Genetic Epidemiology, Department of Preventive Medicine, Keck School of Medicine, University of Southern California/Norris Comprehensive Cancer Center, Los Angeles, CA 90015, USA

[13] Division of Population Health, Health Services Research and Primary Care, University of Manchester, Oxford Road, Manchester, M13 9PL, UK

[14] Division of Public Health Sciences, Fred Hutchinson Cancer Research Center, Seattle, Washington, 98109-1024, USA

[15] Department of Epidemiology, School of Public Health, University of Washington, Seattle, Washington 98195, USA

The PRACTICAL Consortium (http://practical.icr.ac.uk/):

This work was supported by the Canadian Institutes of Health Research, European Commission's Seventh Framework Programme grant agreement n° 223175 (HEALTH-F2-2009-223175), Cancer Research UK Grants C5047/A7357, C1287/A10118, C1287/A16563, C5047/A3354, C5047/A10692, C16913/A6135, and The National Institute of Health (NIH) Cancer Post-Cancer GWAS initiative grant: No. 1 U19 CA 148537-01 (the GAME-ON initiative).

We would also like to thank the following for funding support: The Institute of Cancer Research and The Everyman Campaign, The Prostate Cancer Research Foundation, Prostate Research Campaign UK (now PCUK), The Orchid Cancer Appeal, Rosetrees Trust, The National Cancer Research Network UK, The National Cancer Research Institute (NCRI) UK. We are grateful for support of NIHR funding to the NIHR Biomedical Research Centre at The Institute of Cancer Research and The Royal Marsden NHS Foundation Trust.

The Prostate Cancer Program of Cancer Council Victoria also acknowledge grant support from The National Health and Medical Research Council, Australia (126402, 209057, 251533, , 396414, 450104, 504700, 504702, 504715, 623204, 940394, 614296,), VicHealth, Cancer Council Victoria, The Prostate Cancer Foundation of Australia, The Whitten Foundation, PricewaterhouseCoopers, and Tattersall's. EAO, DMK, and EMK acknowledge the Intramural Program of the National Human Genome Research Institute for their support.

Additional funding and acknowledgments from individual studies in PRACTICAL

COSM
COSM is funded by The Swedish Research Council (grant for the Swedish Infrastructure for Medical Population-based Life-course Environmental Research – SIMPLER), the Swedish Cancer Foundation.

ESTHER
The ESTHER study was supported by a grant from the Baden Württemberg Ministry of Science, Research and Arts.
The ESTHER group would like to thank Hartwig Ziegler, Sonja Wolf, Volker Hermann, Heiko Müller, Karina Dieffenbach, Katja Butterbach for valuable contributions to the study.

KULEUVEN
F.C. and S.J. are holders of grants from FWO Vlaanderen (G.0684.12N and G.0830.13N), the Belgian federal government (National Cancer Plan KPC_29_023), and a Concerted Research Action of the KU Leuven (GOA/15/017). TVDB is holder of a doctoral fellowship of the FWO.

MCC-Spain
"The study was partially funded by the ""Accion Transversal del Cancer"", approved on the Spanish Ministry Council on the 11th October 2007, by the Instituto de Salud Carlos III-FEDER (PI08/1770, PI09/00773-Cantabria, PI11/01889-FEDER, PI12/00265, PI12/01270, PI12/00715, PI15/00069), by the Fundación Marqués de Valdecilla (API 10/09), by the Spanish Association Against Cancer (AECC) Scientific Foundation and by the Catalan Government DURSI grant 2009SGR1489. Samples: Biological samples were stored at the Parc de Salut MAR Biobank (MARBiobanc; Barcelona), which is supported by Instituto de Salud Carlos III FEDER (RD09/0076/00036). Also sample collection was supported by the Xarxa de Bancs de Tumors de Catalunya sponsored by Pla Director d'Oncologia de Catalunya (XBTC). ISGlobal is a member of the CERCA Programme, Generalitat de Catalunya."
We acknowledge the contribution from Esther Gracia-Lavedan in preparing the data. We thank all the subjects who participated in the study and all MCC-Spain collaborators.

PRAGGA
PRAGGA was supported by Programa Grupos Emergentes, Cancer Genetics Unit, CHUVI Vigo Hospital, Instituto de Salud Carlos III, Spain.
PRAGGA wishes to thank Victor Muñoz Garzón, Manuel Enguix Castelo, Sara Miranda Ponte, Carmen M Redondo, Manuel Calaza, Francisco Gude Sampedro, Joaquín González-Carreró and the staff of the Department of Pathology and Biobank of University Hospital Complex of Vigo, Instituto de Investigación Sanitaria Galicia Sur (IISGS), SERGAS, Vigo, Spain; Máximo Fraga, José Antúnez and the Biobank of University Hospital Complex of Santiago, Santiago de Compostela, Spain; and Maria Torres, Angel Carracedo and the Galician Foundation of Genomic Medicine.

SWOG-PCPT/ SWOG-SELECT

PCPT / SELECT is funded by Public Health Service grants U10CA37429 and 5UM1CA182883 from the National Cancer Institute.
The authors thank the site investigators and staff and, most importantly, the participants from PCPT /SELECT who donated their time to this trial.
